# Complement activation in immune thrombotic thrombocytopenic purpura: Possible amplifier and potential therapeutic opportunity

**DOI:** 10.1002/hem3.70508

**Published:** 2026-10-19

**Authors:** Massimo Cugno, Spero R. Cataland, Bernhard Lämmle

**Affiliations:** ^1^ Department of Pathophysiology and Transplantation Università degli Studi di Milano Milan Italy; ^2^ Department of Internal Medicine SC Medicina‐Emostasi e Trombosi, Fondazione IRCCS Ca' Granda Ospedale Maggiore Policlinico Milan Italy; ^3^ Department of Medicine, Division of Hematology Ohio State University Columbus Ohio USA; ^4^ Center for Thrombosis and Hemostasis, University Medical Center Johannes Gutenberg University Mainz Germany; ^5^ University Clinic of Hematology and Central Hematology Laboratory Inselspital, Bern University Hospital, University of Bern Bern Switzerland

Immune thrombotic thrombocytopenic purpura (iTTP) is a paradigmatic antibody‐mediated thrombotic microangiopathy. Its pathophysiological core is well established: inhibitory and/or clearing autoantibodies against ADAMTS13 cause severe ADAMTS13 deficiency, persistence of ultra‐large von Willebrand factor (ULVWF) multimers, platelet adhesion under high shear stress, and platelet‐rich microvascular thrombosis.^1^ This model has transformed diagnosis and therapy. Today, acute iTTP is treated with urgent therapeutic plasma exchange, corticosteroids, increasingly early rituximab, and caplacizumab, which blocks the interaction between VWF and platelet glycoprotein Ib.^2^, ^3^ With these interventions, targeting the dominant pathogenic axis (autoimmunity against ADAMTS13 and ULVWF‐mediated platelet microthrombosis), mortality of acute iTTP has been reduced to below 2%.^4^

Although ADAMTS13 deficiency lies at the center of iTTP pathophysiology, other biological systems that intersect with thromboinflammation may have contributing roles. Complement is one such system. Its activation may amplify endothelial injury, inflammation, renal dysfunction, organ ischemia, and treatment‐refractoriness in selected patients. In addition, it has been hypothesized that complement activation could play a role in long‐term cardiovascular and neurocognitive complications of iTTP. If this is true, the complement system should be investigated as a component of the thromboinflammatory network that determines phenotype and outcome.

## WHY THE COMPLEMENT SYSTEM DESERVES RENEWED ATTENTION IN ITTP

The complement system is increasingly recognized as a bridge between innate immunity, inflammation, endothelial biology, hemostasis, and thrombosis.^5^ Complement activation through the classical, lectin, or alternative pathways converges on C3 cleavage, generation of anaphylatoxins C3a and C5a, opsonization by C3 fragments, and assembly of the terminal complement complex C5b‐9. These mediators are not simply antimicrobial effector molecules. C5a can recruit and activate leukocytes, prime neutrophils, and enhance inflammatory tissue injury.^5^, ^6^ Sublytic C5b‐9 can activate endothelial cells, increase permeability, and promote prothrombotic responses. Such effects are highly relevant to thrombotic microangiopathies, in which endothelial perturbation and microvascular occlusion are central features.^6^ There are at least three reasons why complement activation could be relevant in iTTP.

First, ULVWF is not only a platelet‐adhesive substrate but can also interact with the complement system. In the microcirculation, high shear stress unfolds VWF and exposes platelet‐binding A1 domains, making ULVWF particularly hyperadhesive for platelets. At the same time, endothelial cell‐anchored ULVWF strings can assemble and activate components of the alternative complement pathway, providing a plausible mechanistic link between severe ADAMTS13 deficiency and complement amplification.^7^ Conversely, normal VWF can act as a cofactor for Factor I‐mediated proteolytic inactivation of C3b into iC3b, thereby contributing to complement regulation. ULVWF multimers, however, lack this regulatory cofactor activity, favoring complement activation rather than inhibition.^8^ Thus, the ADAMTS13‐VWF axis and the complement system are biologically connected rather than independent.

Second, animal data support cooperation between ADAMTS13 deficiency and complement activation. Severe ADAMTS13 deficiency alone is often insufficient to reproduce the full human iTTP phenotype in experimental systems, whereas complement activation can synergize with ADAMTS13 deficiency to promote thrombotic microangiopathy.^9^ This observation is consistent with a “multi‐hit” or amplifier model: ADAMTS13 deficiency creates the VWF‐dependent thrombotic substrate, while complement activation contributes to endothelial injury, inflammation, and organ damage.

Third, clinical studies repeatedly show that complement activation is detectable and clinically informative in patients with iTTP. Réti et al. provided one of the first systematic demonstrations of complement activation in TTP, reporting increased C3a and soluble C5b‐9 during acute episodes, with evidence of complement consumption in some patients. The decrease in complement activation products during plasma exchange suggested that complement activation paralleled the acute disease process rather than representing a fixed background abnormality.^10^ Wu and colleagues subsequently linked complement activation with mortality during acute TTP, raising the possibility that complement activation may identify patients with more severe disease biology.^11^ In a later remission study, the same group examined the relationship among ADAMTS13 activity, VWF, and complement activation. Higher soluble C5b‐9, C3a, and C5a levels were associated with detection of ULVWF multimers, suggesting that incomplete restoration of the ADAMTS13‐VWF axis may remain linked to complement activation and vascular vulnerability even outside overt acute microangiopathy.^12^ Westwood et al. further showed that acute antibody‐mediated TTP is associated with both complement and cytokine activation: C3a and C5a were higher during acute disease than remission, and C3a correlated with anti‐ADAMTS13 IgG and IL‐10, placing complement activation within the autoimmune‐inflammatory milieu of iTTP.^13^ Cao et al. connected this process with innate immunity, reporting increased human neutrophil peptides and factor Bb, with Bb correlating with iC3b, soluble C5b‐9, lactate dehydrogenase, and creatinine. Increased iC3b is compatible with increased C3b generation followed by Factor I‐mediated cleavage, implicating alternative pathway activation and neutrophil‐associated inflammation.^14^ Finally, renal dysfunction provides an informative clinical lens: the study by Cugno and colleagues supports the concept that complement activation may be particularly relevant in iTTP patients with renal involvement, a phenotype that may bridge ADAMTS13‐deficient iTTP and complement‐amplified TMA.^15^

All these observations support complement involvement in iTTP. However, a central challenge is interpretation: bystander, biomarker, or pathogenic amplifier? Complement activation in iTTP could be a bystander phenomenon caused by endothelial injury, hemolysis, platelet activation, immune complexes, or ischemia‐reperfusion. It could be a biomarker of disease severity without pathogenic relevance. Finally, it could become an active amplifier that contributes to progression, organ injury, and refractoriness. These possibilities are not mutually exclusive. In many thromboinflammatory disorders, complement activation is both a consequence and a cause of tissue injury.^5^, ^6^ Once activated, complement fragments can amplify leukocyte recruitment, endothelial activation, platelet activation, and microvascular thrombosis. The distinction between “secondary” and “pathogenic” may therefore be too rigid. Even if complement activation were initially triggered by ADAMTS13 deficiency and VWF‐mediated microthrombosis, it may still become biologically important once the thromboinflammatory loop is established. This view distinguishes iTTP from atypical hemolytic uremic syndrome (aHUS). In aHUS, alternative pathway dysregulation is disease‐defining, and complement blockade is foundational therapy. In iTTP, severe ADAMTS13 deficiency remains the disease‐defining basal prothrombotic condition. However, even if secondary to severe ADAMTS13 deficiency, complement activation should not be equated with clinical irrelevance. In iTTP, severe ADAMTS13 deficiency remains the primary pathogenic abnormality, but complement activation may act as an amplifier that is therapeutically meaningful if it contributes to disease severity, organ injury, or delayed response to standard therapy.

## LESSONS FROM OTHER AUTOIMMUNE DISEASES

The therapeutic success of complement inhibition in other autoimmune diseases provides useful insight. These examples illustrate different positions of the complement system in autoimmune disease biology. In cold agglutinin disease, IgM autoantibodies against red blood cells activate the classical pathway, and C1s inhibition with sutimlimab directly interrupts the dominant mechanism of hemolysis.^16^ In anti‐aquaporin‐4‐IgG‐positive neuromyelitis optica spectrum disorder and anti‐acetylcholine receptor antibody‐positive generalized myasthenia gravis, pathogenic autoantibodies activate complement at target tissues, and complement inhibition can prevent antibody‐mediated tissue injury.^17^, ^18^ In ANCA‐associated vasculitis, C5a–C5a receptor signaling contributes to neutrophil activation, and avacopan demonstrates that blocking a specific complement inflammatory axis can be clinically useful without complete terminal pathway blockade.^19^ Although not autoimmune, paroxysmal nocturnal hemoglobinuria provides an additional hematologic model, showing that complement inhibition may be effective at different levels of the cascade, including terminal C5, proximal C3, and the alternative pathway.^20^ Thus, the lesson is not that complement blockade should be broadly transferred to iTTP, but that complement inhibition may be most successful when the relevant pathway, the appropriate patient subset, and the clinically meaningful endpoint are correctly identified.

## THERAPEUTIC IMPLICATIONS: NOT REPLACEMENT, BUT PRECISION ADJUNCT

Complement inhibition should not be proposed as a substitute for established iTTP therapy. Plasma exchange, corticosteroids, rituximab, caplacizumab (with or without plasma exchange), and emerging ADAMTS13 replacement strategies directly target the causal autoimmune and VWF‐dependent mechanisms. Complement blockade, if useful, would likely be an adjunctive or rescue strategy.

The most plausible candidates are patients with refractory iTTP, severe organ involvement, renal‐predominant TTP, or biomarker evidence of intense complement activation. C5 inhibition with eculizumab or ravulizumab could theoretically reduce both C5a and C5b‐9 generation, but current iTTP evidence is limited to eculizumab in case reports.^21^, ^22^

C5a receptor inhibition with avacopan may be attractive when neutrophil activation and inflammatory endothelial injury are prominent, whereas C1s inhibition with sutimlimab would be rational only if immune‐complex‐induced classical pathway activation proved dominant. C3, factor B, or factor D inhibition may be mechanistically appealing when alternative pathway amplification on ULVWF or injured endothelium is central. In this context, one case report described successful use of iptacopan, an oral factor B inhibitor, in combination with plasma exchange, corticosteroids, and rituximab.^23^ Although in this case complement inhibition was successfully used in combination with rituximab, we must consider that rituximab exerts its effect on CD20‐positive B cells through multiple mechanisms, including complement‐dependent cytotoxicity. Thus, if complement inhibition were ever considered in combination with rituximab, it would be prudent to carefully monitor ADAMTS13 activity, anti‐ADAMTS13 antibodies, and B‐cell depletion. The available reports of complement inhibition in iTTP remain case‐specific and hypothesis‐generating; they involved patients with elevated complement activation markers and/or resistance to conventional treatment and none included caplacizumab.^21^, ^22^, ^23^ Given the efficacy of caplacizumab in rapidly interrupting VWF–platelet interaction, future studies should assess whether it also reduces complement activation, thereby helping identify patients with persistent complement activation despite control of the VWF–platelet axis. Such studies should include rigorous patient stratification and longitudinal measurement of complement activation during acute disease, remission, relapse, and treatment response. Candidate biomarkers may include C3a, C5a, soluble C5b‐9, Bb or Ba, iC3b, C4d, C1r/s‐C1 inhibitor complexes, endothelial C5b‐9 deposition, and complement regulatory proteins. Table 1 reports the complement tests most commonly used for clinical practice in patients with iTTP along with the relative analytical and preanalytical aspects. Complement tests should be correlated with ADAMTS13 activity, anti‐ADAMTS13 antibody titers and classes/subclasses, VWF antigen, VWF multimer pattern, platelet count recovery, lactate dehydrogenase, neurologic injury, renal function, exacerbation, relapse, and mortality.

**Table 1:** Analytical and preanalytical aspects of commonly used laboratory tests for the evaluation of the complement system in immune thrombotic thrombocytopenic purpura.

Purpose	Biomarkers	Method	Sampling procedure	Preanalytical considerations
Detect complement system activation.	sC5b‐9	Several ELISA methods are available, and some of them have been approved for diagnostic use in Europe, labeled as CE‐IVD (In Vitro Diagnostic).	Blood samples should be collected by clean venipuncture from an antecubital vein or from a central venous catheter in tubes containing ethylenediaminetetraacetic acid (EDTA) 0.13 mol/L, which chelates calcium and efficiently blocks both complement and coagulation activation. An additional blood sample may be collected in sodium citrate if specific coagulation tests are needed. Within two hours, the tubes should be centrifuged at 2000 × *g* for 10 min at room temperature, and the plasma aliquots are stored in polystyrene tubes at minus 80°C until testing.	For testing sC5b‐9 and C5a, pre‐analytical conditions are critical in order to prevent *in vitro* activation of both complement and coagulation, which are strictly linked and can activate each other. Even a small activation of complement can be detected by these very sensitive methods. An additional blood sample may be collected in sodium citrate if specific coagulation tests are needed.
	C5a	Several ELISA methods are available, and one has been approved for diagnostic use in Europe (CE‐IVD).		
Assess the activation capacity of the complement system after *in vitro* activation.	Percentage of activation of the classical pathway, lectin pathway, and alternative pathway of the complement system.	Immunoenzymatic methods that detect the amount of the terminal complex C5b‐9 generated in serum samples during the activation of the classical, lectin and alternative pathways in the presence of specific activators. They are approved for diagnostic use in Europe (CE‐IVD) and have substituted the old hemolytic assays CH50 and AP50.	For obtaining serum samples, blood samples should be collected by clean venipuncture, allowed to clot, and processed within 2 hours (centrifugation of blood samples and freezing of serum samples).	EDTA is not suitable for functional assays because it blocks complement activation. Serum is commonly used. Although *in vitro* clotting may induce complement activation, if the sample is processed within 2 hours, the *in vitro* activation is trivial and does not interfere with the assay, whereas the *in vivo* consumption and the inherited defect of complement factors are easily detected.
Assess complement consumption or baseline components.	C3	Immunoturbidimetric methods approved for diagnostic use in Europe (CE‐IVD).	Serum is obtained during routine blood sampling.	Interpretation should consider preanalytical clinical conditions such as acute‐phase responses, consumption, plasma exchange, transfusion, and complement‐inhibiting therapy. The use of the same test longitudinally is of help.
	C4			
	Factor H antigen	ELISA methods approved for diagnostic use in Europe (CE‐IVD).		
Detect the presence of autoantibodies to complement inhibitors (usually factor H).	Anti‐factor H autoantibodies (IgG class)	ELISA methods approved for diagnostic use in Europe (CE‐IVD).	Serum is obtained during routine blood sampling.	Anti‐factor H autoantibodies of IgG class are commonly associated with CFHR3/CFHR1 deletion.
Assess genetic alterations of inhibitors and activators of the complement system.	Genetic variants in complement‐related genes: CFH, MCP/CD46, CFI, C3, CFB, THBD, DGKE, CFHR1, CFHR3, and CFHR5	Targeted Next Generation Sequencing (NGS) for CFH, MCP/CD46, CFI, C3, CFB, THBD, DGKE, CFHR1, CFHR3, and CFHR5. Approved for diagnostic use in Europe (CE‐IVD).	Blood samples collected in EDTA are used for DNA extraction and subsequent genetic testing.	Genetic tests are performed when there is suspicion of a defect of inhibitors (loss‐of‐function variants or deletions) or hyperfunction of activators (gain‐of‐function variants).
	CFHR3/CFHR1 deletion	Multiplex ligation‐dependent probe amplification assay (MLPA) to identify CFHR3/CFHR1 deletion.		
		Approved for diagnostic use in Europe (CE‐IVD).		

A practical research agenda would include three steps: (1) observational cohorts to define whether a reproducible “complement activation‐high” iTTP phenotype exists; (2) mechanistic studies to determine whether complement activation is driven mainly by ULVWF, immune complexes, neutrophil activation, endothelial injury, or alternative pathway dysregulation; and (3) small biomarker‐guided interventional trials to test whether targeted complement inhibition, added to current standard therapy, improves clinically meaningful endpoints, including late organ dysfunction sequelae.

## A PROPOSED FRAMEWORK

The most useful framework may be the “ADAMTS13 deficiency‐first, complement‐amplified” model (Figure 1). In this model, severe ADAMTS13 deficiency sets the stage for acute disease by allowing persistence of ULVWF multimers and VWF‐mediated platelet microthrombosis. Complement activation may arise through ULVWF‐dependent alternative pathway amplification, together with additional triggers such as endothelial injury, hemolysis, inflammation, immune complexes, neutrophil activation, and platelet activation. In some patients, this complement activation remains a transient biomarker. In others, it may become a pathogenic amplifier contributing to organ injury, renal dysfunction, refractoriness, or poor long‐term health.

**Figure 1:**
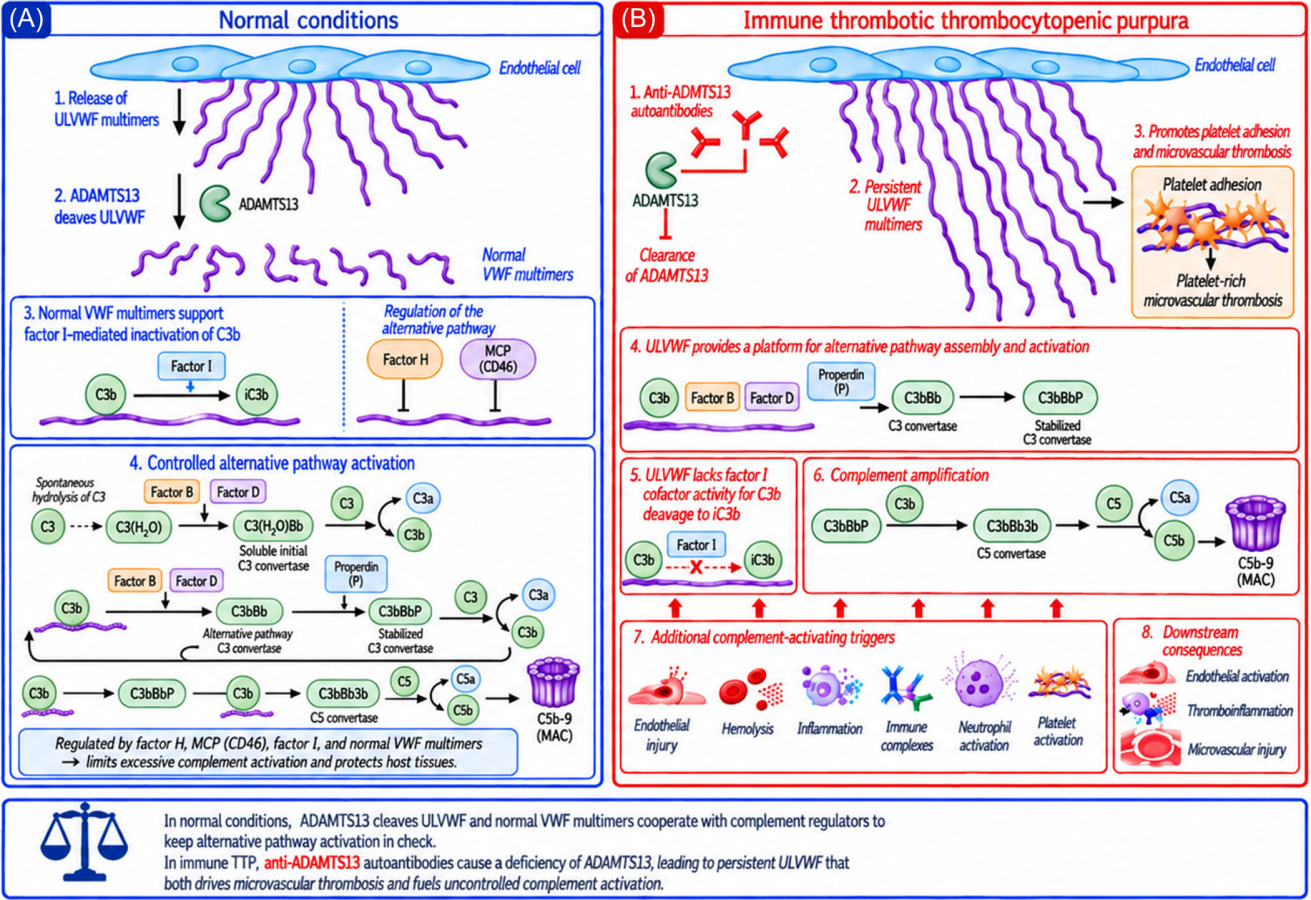
**ADAMTS13‐VWF‐complement interplay in normal conditions (A) and immune thrombotic thrombocytopenic purpura (B).** Under physiological conditions (A), endothelial cell‐released ultra‐large von Willebrand factor (ULVWF) multimers are cleaved by ADAMTS13 into smaller VWF multimers. On endothelial cells, normal VWF multimers can support Factor I‐mediated proteolytic inactivation of C3b into iC3b, limiting the activation of the complement alternative pathway that is also controlled by factor H and membrane cofactor protein (MCP, CD46). The alternative pathway is continuously active at a low level through spontaneous hydrolysis of C3 that binds factor B, which is then cleaved by factor D to form the soluble initial C3 convertase C3(H_2_O)Bb. This enzyme complex can cleave additional C3 molecules, generating C3b that covalently attaches to activating surfaces (pathogen membranes, altered self, or damaged tissue). When bound to a surface, C3b is protected from inhibition, and this bound C3b recruits more factor B and factor D, generating the alternative pathway C3 convertase C3bBb. The stabilizing protein properdin also binds to the alternative pathway C3 convertase, forming a more stable complex, C3bBbP, which can further cleave C3 into C3a and C3b and greatly amplifies the complement activation, which proceeds with the formation of C5 convertase C3bBb3b. C5 convertase cleaves C5 into the anaphylatoxin C5a and the C5b molecule, the latter initiating the formation of the membrane attack complex (MAC; C5b‐9) that leads to the lysis of target cells. In immune thrombotic thrombocytopenic purpura (panel B), anti‐ADAMTS13 autoantibodies inhibit and/or clear ADAMTS13, resulting in persistence of endothelial cell‐secreted and anchored ULVWF multimers. Persistent ULVWF promotes platelet adhesion and platelet‐rich microvascular thrombosis and may also provide a platform for alternative pathway assembly and activation, including C3b, factor B, factor D, and properdin, leading to C3 convertase (C3bBb) formation, generation of C3a and formation of C5 convertase (C3bBb3b) that cleaves C5 into C5a and C5b, which in turn initiates assembly of the terminal complement complex (C5b‐9). Unlike normal VWF multimers, ULVWF multimers lack Factor I cofactor activity for C3b cleavage into iC3b and therefore permit complement amplification. Complement activation in iTTP may also arise through additional triggers, including endothelial injury, hemolysis, inflammation, immune complexes, neutrophil activation, and platelet activation. These convergent mechanisms may amplify endothelial activation, thromboinflammation, and microvascular injury.

This model has a practical consequence: it avoids an overly rigid dichotomy between “TTP is ADAMTS13‐mediated” and “aHUS is complement‐mediated.” Certainly, this dichotomy exists and allows us to define clear disease categories, but it has an important limitation: pathophysiology in individual patients may not strictly follow our diagnostic constructs. Growing evidence indicates interrelationships among ADAMTS13 deficiency, VWF biology, endothelial injury and complement activation. These interactions may play an important role not only in the pathophysiology of iTTP but also in the pathophysiology of other TMAs, particularly those with severe disease or renal dysfunction. Complement blockade is unlikely to be broadly effective in unselected iTTP patients, but may be rational in selected biological phenotypes. Theoretically, patients with low levels of ADAMTS13 activity and elevated markers of complement activation might benefit from complement inhibition therapy both during remission, to prevent further amplification and avert clinical relapse, and during acute attacks, to interrupt complement hyperactivation, a known cause of microvascular damage. Most importantly, the model makes testable predictions: “complement activation‐high” iTTP patients should have more severe organ injury or distinct renal involvement; complement biomarkers should fall with effective disease control; and targeted complement inhibition should improve outcomes mainly in complement biomarker‐enriched subgroups.

## CONCLUSION

Complement activation in iTTP has probably been underestimated because its role as an amplifier within the dominant VWF–platelet mechanism has been difficult to prove. The available evidence supports a cautious but proactive conclusion: complement activation is detectable, biologically plausible, linked to the ADAMTS13‐VWF axis, and associated with inflammation, renal dysfunction, and severe or refractory phenotypes. Predominant renal involvement, in particular, may identify patients in whom complement activation is more prominent, and its inhibition should be specifically investigated.

The field should now move beyond asking whether complement activation occurs in iTTP. It does. The more important questions are when it matters, in whom it matters, how it should be measured, and whether it can be safely and effectively targeted. Answering these questions will require integrated studies of ADAMTS13, VWF, complement system, endothelium, neutrophils, and clinical outcomes. Such studies may identify a subset of patients in whom complement inhibition, added to established therapy, becomes a rational precision‐medicine strategy. Until then, complement blockade in iTTP should remain investigational, but the complement system itself should no longer be presumed to be a marginal bystander.

## AUTHOR CONTRIBUTIONS

**Massimo Cugno**: Conceptualization; writing—original draft; writing—review and editing. **Spero R. Cataland**: Writing—review and editing. **Bernhard Lämmle**: Conceptualization; writing—review and editing.

## CONFLICT OF INTEREST STATEMENT

B.L. was chairman of data monitoring committees (DMCs) of studies investigating recombinant ADAMTS13 for the treatment of congenital and acquired thrombotic thrombocytopenic purpura (Takeda), chairman of the DMC of a study investigating caplacizumab for treatment of autoimmune TTP without plasma exchange (Sanofi); he is on the advisory board of Takeda for TTP. S.R.C. serves as a consultant to Sanofi, Takeda, and Novartis. M.C. declares no conflicts of interest.

## ETHICS STATEMENT

Ethical approval was not required for this work as it was conducted on previously published data.

## FUNDING

This work was partially supported by the Italian Ministry of Health ‐ Bando Ricerca Corrente, but did not receive any specific grant from funding agencies in the public, commercial, or not‐for‐profit sectors. Open access publishing facilitated by Universita degli Studi di Milano, as part of the Wiley ‐ CRUI‐CARE agreement.

## Data Availability

Data sharing is not applicable to this article as no datasets were generated or analyzed during the current study.
